# Low gestational age is associated with less anastomotic complications after open primary repair of esophageal atresia with tracheoesophageal fistula

**DOI:** 10.1186/s12887-020-02170-1

**Published:** 2020-06-03

**Authors:** Carmen Dingemann, Julia Brendel, Julia Wenskus, Sabine Pirr, Nagoud Schukfeh, Benno Ure, Konrad Reinshagen

**Affiliations:** 1 Department of Pediatric Surgery, Hannover Medical School, Carl-Neuberg-Str. 1, 30625 Hannover, Germany; 2 Department of Pediatric Surgery, University Medical Center Hamburg-Eppendorf, Hamburg, Germany; 3 Department of Pediatric Pulmonology, Allergology and Neonatology, Hannover Medical School, Hannover, Germany

**Keywords:** Pediatric surgery, Gestational age, Esophageal atresia, Postoperative outcome, Prematurity, Morbidity, Esophageal anastomosis

## Abstract

**Background:**

The aim of this study was to evaluate anastomotic complications after primary one-staged esophageal atresia (EA) repair relating to the patients` gestational age (GA).

**Methods:**

Retrospective data analyses of patients who underwent closure of tracheoesophageal fistula (TEF) and primary esophageal anastomosis from 01/2007 to 12/2018 in two pediatric surgical centers. Exclusion of EA other than Gross type C, long-gap EA, minimal invasive or staged approach. Postoperative complications during the first year of life were assessed. Associated malformations, the incidence of infant respiratory distress syndrome (IRDS) and intraventricular bleeding were analyzed.

**Results:**

Inclusion of 75 patients who underwent primary EA repair. Low GA was associated with significantly lower incidence of anastomotic complications (*p* = 0.019, *r* = 0.596, 95% CI 0.10–0.85). Incidence of anastomotic leakage (0% vs. 5.5%; *p* = 0.0416), recurrent TEF (0% vs. 5.5%; *p* = 0.0416) und anastomotic stricture (0% vs. 14.5%; *p* = 0.0019) was significantly lower in patients < 34 gestational weeks. Incidence of IRDS (55% vs. 0%; *p* < 0.0001) and intraventricular bleeding (25% vs. 3.6%; *p* = 0.0299) was significantly higher in patients < 34 gestational weeks.

**Conclusions:**

Despite prematurity-related morbidity, low GA did not adversely affect surgical outcome after primary EA repair. Low GA was even associated with a better anastomotic outcome indicating feasibility and safety of primary esophageal reconstruction.

## Background

Since the first successful primary surgical correction of an esophageal atresia (EA) with tracheoesophageal fistula (TEF) in 1941 [1], mortality and morbidity rates have remarkably improved over the last decades [2]. The principle of surgery including an early TEF ligation and division followed by primary or delayed esophageal anastomosis is widely accepted [3, 4].

However, recent advances of perinatal management, neonatal care, technical innovations, surgical expertise, and perioperative concepts have led to increasing numbers of premature low birth weight infants surviving [3, 5–7].

Prematurity, birth weight and/or additional anomalies are usually considered crucial factors in determing the outcome of EA/TEF patients [3, 5, 6, 8, 9]. Nevertheless, the optimal surgical management of this selective group of patients with EA/TEF remains controversial [4, 5, 10–14]. Several authors postulated that staged repair of EA/TEF including primary TEF closure and secondary, delayed esophageal anastomosis in premature infants with low birth weights resulted in a lower rate of anastomotic complications, and thus should be considered the preferred surgical approach in this group of patients [4, 5].

Birth weight (BW), however, has become a less significant determinant for the outcome after EA/TEF repair [15–19]. In obstetric and neonatal practice, the patients´ gestational age (GA) is considered to be a more relevant maturational and physiologic factor than body weight [20–22].

Taking this into account, the aim of this study was to evaluate anastomotic complications such as anastomotic leakage, recurrent fistula, and anastomotic stricture after primary EA/TEF repair in relation to the patients` GA.

## Methods

This study was approved by the Institutional Ethical Review Board of Hannover Medical School, 30,625 Hannover, Germany (approval number 8425_BO_K_2019). A retrospective data analysis of patients with EA and distal TEF (Gross type C) was performed in two tertiary referral centers of pediatric surgery in Germany. The databases of both university hospitals were screened for patients who underwent primary, one-staged, open esophageal reconstruction including TEF ligation and esophageal anastomosis from January 2007 to December 2018. This study includes all patients that underwent initial surgical treatment in either of the two centers, whether they were born in-house or referred by another hospital for further treatment.

Exclusion criteria were incomplete data sets, patients with EA other than Gross type C, long-gap EA, minimal invasive or staged surgical approach resulting in secondary esophageal anastomosis. Long-gap EA was defined as Gross type A and B, but also type C presenting with a long distance between the upper and lower pouch impeding the formation of a primary esophageal anastomosis.

Decision for primary open repair was made according to the selection criteria with regard to the thoracoscopic approach for EA/TEF repair which have been published by our working group [23]. Patient selection *contra* a thoracoscopic approach respectively *pro* an open surgical approach included cardiorespiratory instable patients and/or a BW of < 2000 g. In addition, a lack of required infrastructure or of surgical expertise in the field of thoracoscopic EA/TEF repair also resulted in a decision for open surgery in some cases on public holidays and at weekends.

Furthermore, it is common practice in both participating centers to decide for primary EA/TEF repair in all patients, even in those with low GA. In case of intraoperative complications during primary repair, fistula ligation only might become necessary leading to staged repair.

Endpoints of this study were postoperative anastomotic complications and prematurity-related problems directly related to the patients´ GA. Postoperative complications during the first year of life included anastomotic leakage (diagnosed by contrast study of the esophagus in case of any suspicion), recurrent TEF, and anastomotic stricture (defined as > 3 endoscopic dilatations [24, 25]). Associated anomalies and prematurity-related problems, such as infant respiratory distress syndrome (IRDS) defined by chest radiography and intraventricular bleeding defined by ultrasound [26, 27], were also analyzed.

### Statistical analysis and software

Data were recorded and analyzed in an anonymized form. Incomplete datasets were excluded from statistical analysis (*n* = 4). Infants were categorized according to their GA at birth in either < 34 completed weeks of gestation or ≥ 34 completed weeks of gestation. Means and standard deviations were calculated for continuous variables, frequencies, and percentages for categorical variables. Data were also tested for normal distribution and equality of variances. Differences in clinical endpoints and characteristics were assessed using unpaired *t*-test with Welsh’s correction for unequal variances and sample sizes. Spearman’s rank correlation coefficient was used to discover correlation between GA and postoperative outcome. Differences in the distribution of patients and postoperative outcomes between the two participating university centers were analyzed using Fisher’s exact test and Mann Whitney U test. Data management and statistical analyses were conducted using Excel 2010 (Microsoft Corporation, Redmond, WA, USA) and GraphPad Prism 5 (GraphPad Software, San Diego, CA, USA). Statistical significance was set at the 0.05 level.

## Results

In total, 75 patients with a median GA of 37 [27 + 0/7–41 + 5/7] weeks and a median birth weight of 2550 g [990 g – 4405 g], underwent primary open, one-staged EA/TEF repair from January 2007 to December 2018 spread across the two participating centers. Of these, 20 patients (26.7%) were born at < 34 gestational weeks with a median birth weight of 1305 g [990 g – 2290 g], while 55 patients (73.3%) were born at ≥34 gestational weeks with a median birth weight of 2740 g [1660 g – 4405 g].

Table 1 presents the distribution of the birth weight of our cohort. Approximately half of the included patients (52%) had a normal birth weight (NBW) of ≥2500 g. The remaining patients with birth weights < 2500 g were categorized according to the current standard definition as low birth weight (LBW), very low birth weight (VLBW), and extremely low birth weight (ELBW) [28]. In the group of patients with LBW, the majority (*n* = 15 patients, 68.2%) had a birth weight of 2000-2499 g. There was one patient (1.3%) with an ELBW of 990 g.

**Table 1:** Distribution of body weight at birth (*n* = 75)

Body weight at birth [g]	Classification of birth weight	No. of patients [%]
**≤999**	*Extremely low birth weight (ELBW)*	**1 (1%)**
		1 (1%) < 34 GA
		0 ≥ 34 GA
**1000–1499**	*Very low birth weight (VLBW)*	**13 (17%)**
		13 (17%) < 34 GA
		0 ≥ 34 GA
**1500–2499**	*Low birth weight (LBW)*	**22 (29%)**
		6 (8%) < 34 GA
		16 (21%) ≥34 GA
**≥2500**	*Normal birth weight (NBW)*	**39 (52%)**
		0 < 34 GA
		39 (52%) ≥34 GA

In total, 36 patients (48%) were diagnosed with associated anomalies (7 patients < 34 gestational weeks, 29 patients ≥34 gestational weeks) [Table 2]. Every fifth patient had a documented VACTERL association. Patients ≥34 gestational weeks had a significantly higher rate of associated anomalies when compared to patients with lower GA.

**Table 2:** Associated anomalies (multiple selections per patient possible)

Comorbidity	No. of all patients (***n*** = 75)	No. of patients < 34 GA (***n*** = 20)	No. of patients ≥ 34 GA (***n*** = 55)	***P*** value
VACTERL	16 (21.3%)	2 (10%)	14 (25.5%)	0.0477
Vertebral	4 (5.3%)	0 (0%)	4 (7.3%)	0.0222
Cardiac (with hemodynamic relevance)	18 (24%)	2 (10%)	16 (29%)	0.0221
Anorectal	7 (9.3%)	0 (0%)	7 (12.7%)	0.0350
Renal	13 (17.3%)	3 (15%)	10 (18.2%)	0.3728
Limb	3 (4%)	0 (0%)	3 (5.5%)	0.0416
Chromosomal aberration	2^a^ (2.7%)	0 (0%)	2 (3.6%)	0.0796
Other	14 (18.7%)	5 (25%)	9 (16.4%)	0.2222

^a^One patient (1.3%) was diagnosed with trisomy 21, one patient (1.3%) was diagnosed with a partial trisomy 18

Out of the 20 patients who were born at < 34 gestational weeks, 6 patients (30%) were operated by a head of department, 14 patients (70%) were operated by a consultant. In the group of 55 patients who were born ≥34 gestational weeks, 14 patients (25%) were operated by a head of department, 35 patients (64%) were operated by a consultant, 5 patients (9%) were operated by a specialized registrar, and one patient (2%) was operated by a trainee.

Moreover, the relation between operating surgeon and anastomotic complications was assessed: The 3 patients who presented with anastomotic leakage were operated by either a head of department (*n* = 1) or a consultant (*n* = 2). The 3 patients who were diagnosed with recurrent fistula were either operated by a head of department (*n* = 1) or a consultant (n = 2). The 8 patients who presented with anastomotic stricture were operated either by a head of department (*n* = 1) or a consultant (*n* = 7). The 6 patients (8%) who were operated by either a specialized registrar (*n* = 5) or a trainee (n = 1) did not present with any anastomotic complication.

**Fig. 1:**
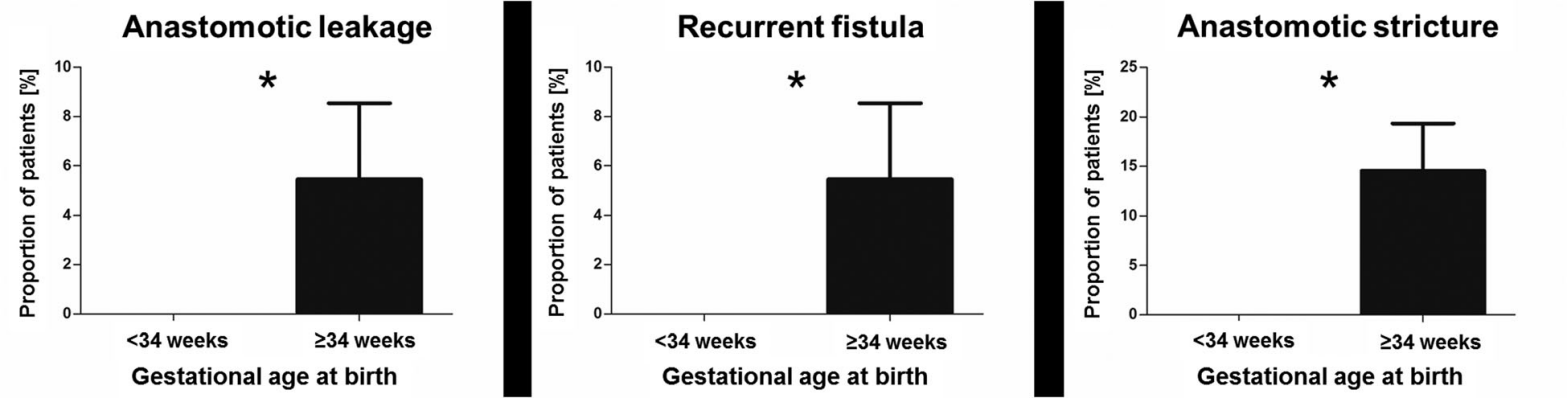
Postoperative complications after primary esophageal anastomosis for esophageal atresia with distal tracheoesophageal fistula; (*p < 0.05, unpaired t-test with Welsh’s correction)

The incidence of postoperative anastomotic complications, such as anastomotic leakage (0% vs. 5.5%; *p* = 0.0416) [Fig. 1], recurrent TEF (0% vs. 5.5%; *p* = 0.0416) [Fig. 1], and anastomotic stricture (0% vs. 14.5%; *p* = 0.0019) [Fig. 1] was significantly lower in patients < 34 gestational weeks compared to patients ≥34 gestational weeks [Table 3].

**Table 3:** Postoperative outcome after primary esophageal anastomosis for esophageal atresia with distal tracheoesophageal fistula

Complications		No. of patients < 34 GA (***n*** = 20)	No. of patients ≥ 34 GA (***n*** = 55)
Related to surgical intervention	Anastomotic leakage	**0**	3* (5.5%)^**a**^
	Recurrent fistula	**0**	3* (5.5%)^**b**^
	Anastomotic stricture	**0**	8* (14.5%)^**c**^
Related to prematurity	Infant respiratory distress syndrome	11* (55%)	**0**
	Intraventricular bleeding	5* (25%)	**2 (3.6%)**

*p < 0.05

^a^Incidence of anastomotic leakage reported in literature: 11–16% [2]

^b^Incidence of recurrent fistula reported in literature: 4–9% [2]

^c^Incidence of anastomotic stricture reported in literature: 25–38% [2]

**Fig. 2:**
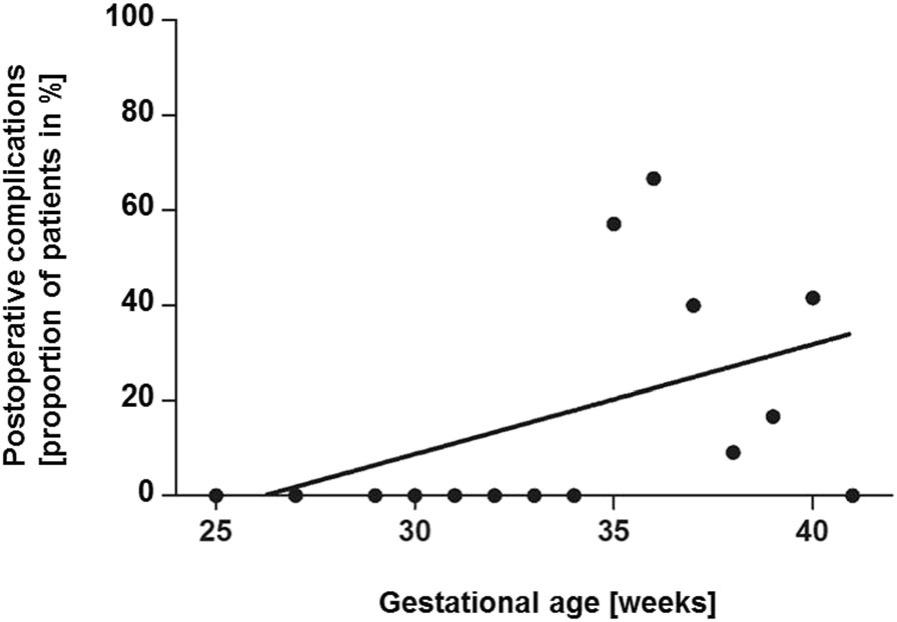
Correlation between incidence of pooled postoperative complications (anastomotic leakage, recurrent fistula, anastomotic stricture) and gestational age (in weeks); (Spearman’s correlation, *p* = 0.019, *r* = 0.596, 95% CI 0.10–0.85)

Moreover, a low GA was associated with a significantly lower incidence of pooled postoperative anastomotic complications (anastomotic leakage + recurrent fistula + anastomotic stricture) in the whole collective of patients (*p* = 0.019, *r* = 0.596, 95% CI 0.10–0.85) [Fig. 2].

Anastomotic leakage was treated conservatively with antibiotics in two cases; in one case, chest drainage was placed. All three patients with recurrent TEF underwent revisional surgery for fistula ligation. Anastomotic stricture was treated with endoscopic dilatations in all cases (median 5 [3–19] dilatations).

Prematurity-related complications such as IRDS (55% vs. 0%; *p* < 0.0001) and intraventricular bleeding (25% vs. 3.6%; *p* = 0.0299) were found to be significantly higher in patients < 34 gestational weeks [Table 3]. There were no significant differences between both GA groups with regard to the incidence of tracheomalacia and gastroesophageal reflux disease (data not shown). There was no postoperative mortality.

There was an equal distribution of included patients and reported anastomotic complications between the two participating university centers: In center A, 11 EA/TEF patients < 34 gestational weeks were included, in center B, 9 EA/TEF patients < 34 gestational weeks were included (center A 35% vs. center B 20%; *p* = 0.188). In center A, 9 patients and in center B, 5 patients presented with anastomotic complications. This has been proven for the overall complication rate (center A: 64% vs. center B: 36%; *p* = 0.25), but also separately for each of the anastomotic complications [anastomotic leakage: 66.7% vs. 33.3%; *p* = 0.187; recurrent fistula: 0% vs. 100%; *p* = 0.241; anastomotic stricture: 62% vs. 38%; *p* = 0.103].

## Discussion

### Prognostic classification systems

During the past decades, multiple authors introduced various prognostic classification systems regarding outcome and survival of EA patients [3, 17, 29–31]. Birth weight has consistently been reported as a significant determinant of overall survival [17]. In 1962, Waterston et al. proposed the first prognostic classification based on birth weight, associated anomalies and pneumonia [31]. In 1993, Poenaru et al. reported that birth weight was not found to independently influence mortality resulting in a novel “Montreal classification” based on two other prognostic factors, namely pre-operative ventilator dependence and associated anomalies [29]. One year later, Spitz et al. revised the original Waterston classification by focussing on birth weight and major congenital heart disease [30]. Since then, several attempts to revise these classifications have been made with birth weight becoming a less significant determinant for survival, as compared to the Waterston and Spitz series [4, 15–19].

However, despite improvements in survival ranging from 36% before 1950s to as high as ≥95% since 1995, the incidence of anastomotic complications and postoperative morbidity remains consistently high [2, 4, 32].

It has recently been postulated that neither surgeon nor hospital volume significantly impacted outcomes after EA/TEF repair in a study including more than 3000 neonates [33]. Nonetheless, there is general acceptance that surgical repair of an esophageal atresia should be only performed if suitable expertise and infrastructure is available. In this study, the vast majority of patients (92%) were operated by most experienced surgeons (either head of department or consultant). Anastomotic complications were documented without exception in patients who were operated by experienced surgeons, and not by trainees. For this reason, a potential bias due to the expertise of the operating surgeon can be ruled out – all the more in the light of the current literature [33].

### Surgical approaches for EA/TEF repair

Different surgical approaches have been proposed for EA/TEF repair in preterm and low birth weight infants, such as one-staged, delayed and staged repair [4, 5, 11, 13, 14]. Advantages of primary EA/TEF repair are undisputably recognized, such as early esophageal continuity, decreased risk of aspiration, lesser feeding dysfunction, and avoidance of further interventions [3, 4, 11, 14]. In contrast, it has been postulated that extensive dissection of the esophageal segments aiming to achieve primary anastomosis in preterm infants may result in significantly higher anastomotic complications due to premature tissues and potential ischemia compared to full-term neonates [4, 5, 13, 14]. Consequently, the optimal timing of esophageal anastomosis in preterm patients remains controversial [5, 11].

Prematurity is largely associated with low birth weight. Therefore, the postoperative outcome of EA/TEF patients has been commonly evaluated relating to body weight at birth [3, 5, 10, 11, 13, 14]. Seitz et al. supported the surgical approach of a primary anastomosis in VLBW or ELBW infants with EA/TEF in the absence of other significant anomalies [11]. However, this statement was based on a small series of only four cases [11]. Schmidt et al. observed a slightly lower incidence of anastomotic leakage and strictures in ELBW and VLBW infants after primary repair [10]. Conversely, Petrosyan et al. concluded from their series of 25 included cases with birth weights < 1500 g that delayed repair resulted in a lower rate of anastomotic complications and overall morbidity compared to primary repair [4]. Moreover, Alexander et al. promoted the approach of a staged repair in neonates weighing < 2000 g based on a series of 25 cases with a higher incidence of postoperative complications in those who had undergone primary repair [13].

In contrast to the above mentioned studies focussing on body weight at birth, a recent risk analysis could not find any correlation between birth weight and anastomotic complications after EA/TEF repair [12].

### Gestational age

Prematurity is defined by the use of GA [34, 35]. Obstetric and neonatal practice consider 34 completed weeks of gestation a maturational and physiological milestone in prematurity [20, 36]. This assertion for instance is supported by the guidelines compliant corticosteroid administration before anticipated preterm birth as one of the most important antenatal therapies available to improve newborn outcomes [37]: The administration of antenatal corticosteroids is recommended for fetuses with a GA < 34 weeks [38]. After this threshold, postnatal complications such as respiratory distress, brain hemorrhage or bowel perforation occur far less than in babies born before 34 gestational weeks [36]. Based on this knowledge, the patient collective of the present study was allocated according to their GA at birth in either < 34 completed weeks of gestation or ≥ 34 completed weeks of gestation.

Considering the contradictory results of the publications cited above with regard to postoperative outcome in relation to the patients´ weight, this study focussed on the impact of the patients´ GA rather than weight on the anastomotic outcome after EA/TEF repair.

The incidence of anastomotic complications in our patient collective is in line or rather on a low level compared to the current literature [2]. Furthermore, patients with a low GA even presented with a significant superior anastomotic outcome [Table 3].

### Associated anomalies & prematurity-related problems

It is widely accepted, that associated anomalies and prematurity-related problems significantly affect the morbidity and mortality of EA patients [3, 5, 10].

In this study, every fifth patient presented with a VACTERL association which is considerably higher than rates reported in the current literature [6, 8]. Nonetheless, the overall incidence of associated anomalies was even lower in our cohort compared to the reported numbers in literature [6, 12, 39, 40], but specific comorbidities have been found to be significantly higher in the group of patients ≥34 gestational weeks compared to the group of patients < 34 gestational weeks, such as hemodynamically relevant cardiac anomalies [Table 2]. In our cohort, 29% of patients ≥34 gestational weeks with hemodynamically relevant cardiac anomalies underwent open surgery which might have undergone a thoracoscopic repair otherwise according to the applied selection criteria. This might lead to a potential bias. Nonetheless, 71% of patients ≥34 gestational weeks underwent open repair although they did not present with relevant associated anomalies. In these cases, decision for the open approach was based on timing (EA/TEF repair on public holidays or at weekends) and/or individual surgeon’s preference or expertise.

Therefore, the favorable postoperative anastomotic outcome of patients with a lower GA cannot adequately be explained by the smaller number of cardiac anomalies. All the more, the superior outcome cannot be sufficiently declared by the absence of other associated anomalies such as vertebral or anorectal malformations as they have not been proven to affect the esophageal anastomosis.

Notwithstanding the above, Borruto et al. demonstrated in a recent meta-analysis that the anastomotic outcome did not differ between the open and thoracoscopic EA/TEF repair [41].

Our results further indicate that prematurity-related problems such as IRDS and intraventricular bleeding were significantly higher in patients < 34 gestational weeks which is consistent with the available literature [26, 27].

In summary and according to our findings, EA/TEF patients with a low GA demonstrated significantly better outcomes of the esophageal anastomosis, while significantly suffering from prematurity-related complications.

Fundamentally, the authors wish to highlight that comparative studies evaluating one-staged vs. delayed vs. staged EA/TEF repair are urgently needed to fully capture the anastomotic outcome of patients with low GA compared to term infants.

### Limitations

The authors are aware of the relatively small number of included patients. However, considering the low incidence of this congenital condition [42, 43] and the even lower incidence of EA/TEF patients which are born preterm, to the best of our knowledge, this study provides the largest series of patients in this respect. In this patient collective, specific results were shown to be significant. Therefore, it is tempting to extrapolate these results to bigger sample sizes. Moreover, the authors are aware of the limitations of the retrospective nature of this study.

This study includes data sets of patients from two university centers of pediatric surgery which impedes standardization of results. However, the ultimate objective of this study was to minimize the heterogeneity of the patient collective in order to ensure the best possible comparability of the groups with regard to the anastomotic outcome. Therefore, only a meticulously defined group of patients has been included (only Gross type C, only short-gap EA, only open surgical approach, only one-staged repair) which may also result in a certain bias.

## Conclusions

Previous studies on the surgical outcome after EA/TEF repair widely focused on the association between body weight and anastomotic complications. To the best of our knowledge, this is the first study introducing the patients´ gestational age rather than body weight in the light of postoperative complications such as anastomotic leakage, recurrent fistula and anastomotic stricture. Prematurity was associated with higher neonatal morbidity such as IRDS and intraventricular bleeding. However, low gestational age was associated with a significantly lower incidence of anastomotic complications after one-staged EA/TEF repair. Our data suggests that primary esophageal reconstruction is feasible and safe in patients with low gestational age. Moreover, gestational age is a reliable determinant of the outcome of esophageal anastomosis.

## Data Availability

The datasets generated during and/or analyzed during the current study are not publicly available due to strict data protection guidelines, but are available from the corresponding author on reasonable request.

## Abbreviations

EA: Esophageal atresia
TEF: Tracheoesophageal fistula
GA: Gestational age
IRDS: Infant respiratory distress syndrome
NBW: Normal birth weight
LBW: Low birth weight
VLBW: Very low birth weight
ELBW: Extremely low birth weight
VACTERL: (V) = vertebral abnormalities; (A) = anal atresia; (C) = cardiac defects; (T) = tracheal anomalies; (E) = esophageal atresia; (R) = renal and radial abnormalities; (L) = limb abnormalities

## References

[1] Haight C, Towsley HA (1943). Congenital atresia of the oesophagus with trachea-oesophageal fistula Extrapleural ligation of fistula and end-toend-anastomosis of oesophageal segments. Surg Gynecol Obstet.

[2] Zimmer J, Eaton S, Murchison LE, de Coppi P, Ure BM, Dingemann C (2019). State of play: eight decades of surgery for esophageal atresia. Eur J Pediatr Surg.

[3] Hannon EJ, Billington J, Kiely EM, Pierro A, Spitz L, Cross K (2016). Oesophageal atresia is correctable and survivable in infants less than 1 kg. Pediatr Surg Int.

[4] Petrosyan M, Estrada J, Hunter C, Woo R, Stein J, Ford HR (2009). Esophageal atresia/tracheoesophageal fistula in very low-birth-weight neonates: improved outcomes with staged repair. J Pediatr Surg.

[5] Zani A, Wolinska J, Cobellis G, Chiu PPL, Pierro A (2016). Outcome of esophageal atresia/tracheoesophageal fistula in extremely low birth weight neonates (<1000 grams). Pediatr Surg Int.

[6] Malakounides G, Lyon P, Cross K, Pierro A, De Coppi P, Drake D (2016). Esophageal atresia: improved outcome in high-risk groups revisited. Eur J Pediatr Surg.

[7] Goldenberg RL, Culhane JF (2007). Low birth weight in the United States. Am J Clin Nutr.

[8] Peters RT, Ragab H, Columb MO, Bruce J, MacKinnon RJ, Craigie RJ (2017). Mortality and morbidity in oesophageal atresia. Pediatr Surg Int.

[9] Yamoto M, Nomura A, Fukumoto K, Takahashi T, Nakaya K, Sekioka A (2018). New prognostic classification and managements in infants with esophageal atresia. Pediatr Surg Int.

[10] Schmidt A, Obermayr F, Lieber J, Gille C, Fideler F, Fuchs J (2017). Outcome of primary repair in extremely and very low-birth-weight infants with esophageal atresia/distal tracheoesophageal fistula. J Pediatr Surg.

[11] Seitz G, Warmann SW, Schaefer J, Poets CF, Fuchs J (2006). Primary repair of esophageal atresia in extremely low birth weight infants: a single-center experience and review of the literature. Biol Neonate.

[12] Okata Y, Maeda K, Bitoh Y, Mishima Y, Tamaki A, Morita K (2016). Evaluation of the intraoperative risk factors for esophageal anastomotic complications after primary repair of esophageal atresia with tracheoesophageal fistula. Pediatr Surg Int.

[13] Alexander F, Johanningman J, Martin LW (1993). Staged repair improves outcome of high-risk premature infants with esophageal atresia and tracheoesophageal fistula. J Pediatr Surg.

[14] Pohlson EC, Schaller RT, Tapper D (1988). Improved survival with primary anastomosis in the low birth weight neonate with esophageal atresia and tracheoesophageal fistula. J Pediatr Surg.

[15] Turner B, Dasgupta R, Brindle ME (2014). A contemporary prediction rule for esophageal atresia (EA) and tracheo-esophageal fistula (TEF). J Pediatr Surg.

[16] Okamoto T, Takamizawa S, Arai H, Bitoh Y, Nakao M, Yokoi A (2009). Esophageal atresia: prognostic classification revisited. Surgery..

[17] Choudhury SR, Ashcraft KW, Sharp RJ, Murphy JP, Snyder CL, Sigalet DL (1999). Survival of patients with esophageal atresia: influence of birth weight, cardiac anomaly, and late respiratory complications. J Pediatr Surg.

[18] Deurloo JA, Ekkelkamp S, Schoorl M, Heij HA, Aronson DC (2002). Esophageal atresia: historical evolution of management and results in 371 patients. Ann Thorac Surg.

[19] Calisti A, Oriolo L, Nanni L, Molle P, Briganti V, D'Urzo C (2004). Mortality and long term morbidity in esophageal atresia: the reduced impact of low birth weight and maturity on surgical outcome. J Perinat Med.

[20] Cunningham FG, Williams JW. Williams obstetrics. 2nd ed. New York, NY: McGraw-Hill; 2005.

[21] Bakewell-Sachs S, Medoff-Cooper B, Escobar GJ, Silber JH, Lorch SA (2009). Infant functional status: the timing of physiologic maturation of premature infants. Pediatrics..

[22] Raju TNK, Higgins RD, Stark AR, Leveno KJ (2006). Optimizing care and outcome for late-preterm (near-term) infants: a summary of the workshop sponsored by the National Institute of Child Health and Human Development. Pediatrics..

[23] Dingemann C, Zoeller C, Ure B (2013). Thoracoscopic repair of oesophageal atresia: results of a selective approach. Eur J Pediatr Surg.

[24] Dingemann C, Eaton S, Aksnes G, Bagolan P, Cross KM, De Coppi P, et al. ERNICA Consensus Conference on the Management of Patients with Esophageal Atresia and Tracheoesophageal Fistula: Follow-up and Framework. Eur J Pediatr Surg. 2019Epub ahead of print. 10.1055/s-0039-3400284. 10.1055/s-0039-3400284 31777030

[25] Krishnan U, Mousa H, Dall'Oglio L, Homaira N, Rosen R, Faure C (2016). ESPGHAN-NASPGHAN guidelines for the evaluation and treatment of gastrointestinal and nutritional complications in children with esophageal atresia-Tracheoesophageal fistula. J Pediatr Gastroenterol Nutr.

[26] Turitz AL, Gyamfi-Bannerman C (2017). Comparison of respiratory outcomes between preterm small-for-gestational-age and appropriate-for-gestational-age infants. Am J Perinatol.

[27] *EuroNeoStat Annual Report for Very Low Gestational Age Infants 2014. The ENS Project.* Hospital de Cruces, Unidad Neonatal 5-D, Plaza de Cruces s/n, 48903 Barakaldo, Spain. Available at: webgate.ec.europa.eu/chafea_pdb/assets/files/pdb/20133306/20133306_d07-00_ps_en_agreed_public_contents.pdf. Accessed January 17, 2020.

[28] P07 - Disorders related to short gestation and low birth weight in ICD-10. Available at: https://icd.who.int/browse10/2016/en#/P07. Accessed January 17, 2020.

[29] Poenaru D, Laberge JM, Neilson IR, Guttman FM (1993). A new prognostic classification for esophageal atresia. Surgery..

[30] Spitz L, Kiely EM, Morecroft JA, Drake DP (1994). Oesophageal atresia: at-risk groups for the 1990s. J Pediatr Surg.

[31] Waterston DJ, Bonham-Carter RE, Aberdeen E (1963). Congenital tracheo-oesophageal fistula in association with oesophageal atresia. Lancet.

[32] Engum SA, Grosfeld JL, West KW, Rescorla FJ, Scherer LR (1995). Analysis of morbidity and mortality in 227 cases of esophageal atresia and/or tracheoesophageal fistula over two decades. Arch Surg.

[33] Lawrence AE, Minneci PC, Deans KJ, Kelley-Quon LI, Cooper JN (2019). Relationships between hospital and surgeon operative volumes and outcomes of esophageal atresia/tracheoesophageal fistula repair. J Pediatr Surg.

[34] Silverman WA (1967). American academy of pediatrics. Committee on fetus and newborn. Nomenclature for duration of gestation, birth weight and intra-uterine growth. Pediatrics..

[35] Kramer MS, Papageorghiou A, Culhane J, Bhutta Z, Goldenberg RL, Gravett M (2012). Challenges in defining and classifying the preterm birth syndrome. Am J Obstet Gynecol.

[36] Fleischmann AR (2016). Pediatric Ethics: Protecting the Interests of Children: Oxford University press.

[37] No CO (2017). 713: antenatal corticosteroid therapy for fetal maturation. Obstet Gynecol.

[38] Schmitz T, Sentilhes L, Lorthe E, Gallot D, Madar H, Doret-Dion M (2019). Preterm premature rupture of the membranes: guidelines for clinical practice from the French College of Gynaecologists and Obstetricians (CNGOF). Eur J Obstet Gynecol Reprod Biol.

[39] Bogs T, Zwink N, Chonitzki V, Hölscher A, Boemers TM, Münsterer O (2018). Esophageal atresia with or without Tracheoesophageal fistula (EA/TEF): Association of Different EA/TEF subtypes with specific co-occurring congenital anomalies and implications for diagnostic workup. Eur J Pediatr Surg.

[40] Sfeir R, Michaud L, Sharma D, Richard F, Gottrand F (2015). National Esophageal Atresia Register. Eur J Pediatr Surg.

[41] Borruto FA, Impellizzeri P, Montalto AS, Antonuccio P, Santacaterina E, Scalfari G (2012). Thoracoscopy versus thoracotomy for esophageal atresia and tracheoesophageal fistula repair: review of the literature and meta-analysis. Eur J Pediatr Surg.

[42] Wang B, Tashiro J, Allan BJ, Sola JE, Parikh PP, Hogan AR (2014). A nationwide analysis of clinical outcomes among newborns with esophageal atresia and tracheoesophageal fistulas in the United States. J Surg Res.

[43] Sfeir R, Bonnard A, Khen-Dunlop N, Auber F, Gelas T, Michaud L (2013). Esophageal atresia: data from a national cohort. J Pediatr Surg.

